# Minimising violence and restrictive practices within acute inpatient psychiatric wards

**DOI:** 10.1192/j.eurpsy.2023.2184

**Published:** 2023-07-19

**Authors:** M. Firdosi, C. Wahoviak, T. John, A. Kemp, D. Lagadu, A. Qazi

**Affiliations:** 1 Kent and Medway NHS & Social Care Partnership Trust, Kent, United Kingdom; 2 Quality Improvement, Kent and Medway NHS & Social Care Partnership Trust, Kent, United Kingdom

## Abstract

**Introduction:**

The number of incidences of violence and restrictive practices within acute inpatient psychiatric wards are significantly high which makes these units less conducive for recovery and less therapeutic. Staff and patient survey results highlighted their concerns and their desire to have a safe environment to work and a less volatile therapeutic environment.

**Objectives:**

The aim of this QI project was to reduce violence and restrictive practices within acute inpatient units.

**Methods:**

PDSA cycle was used to achieve the objective.

**Plan:**

Primary and secondary drivers were identified and were illustrated using driver diagram. Three units were identified for pilot study. The group has agreed to collate change ideas from service users and restraint data from internal system will be used to review the impact of changes.

**Do:**

Meeting were conducted with service users from these units to populate change ideas. Additionally, the Acute care group also outlined some practice related change ideas such as enhanced recruitment of substantive staff, safety pods, introducing safety huddles, revising therapeutic planner, developing safe care champions and inclusion of professionals from various disciplines such as drama therapist, sports technicians and peer support workers that are traditionally not included in MDT. The change ideas were implemented in one of the selected units.

**Study:**

The group reviewed the feasibility of change ideas and agreed on change ideas that got more support from service users which were projectors to play music, soothing DVDs to assist with relaxation and ear defenders.

**Action:**

All change ideas were implemented on the pilot units.

**Results:**

The QI project has enabled the trust to reduce the number of violence and restrictive practices on all the three units, with a team approach and using a multipronged approach, co-production and openness key to positive results.

In due course we also liaised with the wards to get qualitative feedback from the service users to see how they felt about this new change.

A year after the initial data was collected, to see if there had been the intended 50 per cent reduction in violence and restrictive practices. The team were delighted to find that they had exceeded this aim with a 56 per cent reduction with only 12.3 incidents being reported over the 3-month period.

**Conclusions:**

The QI project on pilot wards have enabled to reduce the number of violence and restrictive practices on all the three units.

Team approach and using a multipronged approach, coproduction and openness key to positive results.

The next step is to implement these change ideas on all other units and looking into the economic value and saving as part of this project, given lesser incidents and staff requirements.

**Disclosure of Interest:**

None Declared

